# Effects of multi-domain lifestyle interventions on sarcopenia measures and blood biomarkers: secondary analysis of a randomized controlled trial of community-dwelling pre-frail and frail older adults

**DOI:** 10.18632/aging.202705

**Published:** 2021-03-19

**Authors:** Yanxia Lu, Mathew Niti, Keng Bee Yap, Crystal Tze Ying Tan, Ma Shwe Zin Nyunt, Liang Feng, Boon Yeow Tan, Gribson Chan, Sue Anne Khoo, Sue Mei Chan, Philip Yap, Anis Larbi, Tze Pin Ng

**Affiliations:** 1 Department of Medical Psychology and Ethics, School of Basic Medical Sciences, Shandong University, Jinan 250012, China; 2 Performance and Technology Assessment Department, Ministry of Health, Singapore; 3 Geriatric Medicine and Palliative Medicine Department, Ng Teng Fong General Hospital, Singapore; 4 Biology of Ageing Laboratory, Singapore Immunology Network (SIgN), Agency for Science Technology and Research (A*STAR), Immunos Building, Biopolis, Singapore; 5 Gerontology Research Programme, Department of Psychological Medicine, National University Health System, Yong Loo Lin School of Medicine, National University of Singapore, Singapore; 6 Medical Services Department, St Luke’s Hospital, Singapore; 7 Rehabilitation Services Division, St Luke’s Hospital, Singapore; 8 Psychological Medicine Department, Khoo Teck Puat Hospital, Singapore; 9 Nutrition and Dietetics Department, Khoo Teck Puat Hospital, Singapore; 10 Geriatric Medicine Department, Khoo Teck Puat Hospital, Singapore; 11 Geriatrics Division, Department of Medicine, Research Center on Aging, University of Sherbrooke, Sherbrooke, Quebec, Canada

**Keywords:** sarcopenia, lifestyle intervention, randomized controlled trial, inflammation, homeostasis

## Abstract

Few studies have comprehensively described changes in blood biomarkers of the physiological responses underlying sarcopenia reduction associated with lifestyle interventions. In this study, we performed secondary analyses of data in a randomized controlled trial of multi-domain lifestyle interventions (6-month duration physical exercise, nutritional enrichment, cognitive training, combination and standard care control) among 246 community-dwelling pre-frail and frail elderly, aged ≥65 years, with and without sarcopenia. Appendicular lean mass (ALM), lower limb strength, gait speed, and blood levels of markers of muscle metabolism, inflammation, anti-oxidation, anabolic hormone regulation, insulin signaling, tissue oxygenation were measured at baseline, 3-month and 6-month post-intervention. Multi-domain interventions were associated with significant (p < 0.001) reduction of sarcopenia at 3-month and 6-month post-intervention, improved gait speed, enhanced lower limb strength, and were equally evident among sarcopenic participants who were slower at baseline than non-sarcopenic participants. Active intervention was associated with significantly reduced inflammation levels. Sarcopenia status and reduction were associated with blood biomarkers related to muscle metabolism, steroid hormone regulation, insulin-leptin signaling, and tissue oxygenation. Physical, nutritional and cognitive intervention was associated with measures of sarcopenia reduction, together with changes in circulating biomarkers of anabolic and catabolic metabolism underlying sarcopenia.

## INTRODUCTION

The aging process is characterized by a dramatic decline in lean body or muscle mass over the decades of life and the accelerated loss of strength and function that are the hallmarks of sarcopenia [1]. The multiple adverse outcomes of sarcopenia include falls, multi-morbidity, impaired quality of life, disability and mortality [2]. As sarcopenia is potentially reversible, its effective treatment can have a dramatic impact on reducing the disease burden and increasing healthy lifespan of older people.

Age-related anabolic resistance -a blunted synthetic response to protein and exercise, are primary drivers of muscle mass loss in the aging process [3, 4]. Interventions targeting physical inactivity and malnutrition which are the primary causes of sarcopenia can thus potentially improve muscle quantity and quality and prevent or delay the progression of sarcopenia [5]. Clinical trial studies show that the effect of conventional nutritional interventions alone on muscle mass and strength in sarcopenic elderly subjects are limited [6, 7], but recent studies suggest that specific nutritional supplements such as leucine-enriched whey protein and vitamin D may increase muscle mass and muscle function in sarcopenic and malnourished older patients [8–11]. Physical exercise alone [12–14] or with nutritional intervention [15–25] are consistently shown to improve muscle mass, strength and gait speed in older adults with sarcopenia. Cognitive performance and decline are reportedly associated with handgrip strength and sarcopenia in older persons [26], and cognitive training has been shown to maintain and improve gait speed and balance [27, 28], but its effect on sarcopenia is largely unrecognized.

There is a paucity of studies that have explored changes in blood biomarkers of the physiological responses underlying sarcopenia reduction associated with lifestyle interventions. Chronic inflammation is regarded as a major pathophysiological mechanism underlying sarcopenia and has been investigated most commonly in lifestyle interventions for sarcopenia reduction [18, 29]. Exercise intervention reportedly reduce tumor necrosis factor alpha (TNF-α) mRNA and protein levels, while improving muscle strength in frail elderly [29]. Whey protein, amino acids, and vitamin D supplementation are reported to lower C-reactive protein (CRP) level in sarcopenic elderly [18]. Soy protein supplementation and exercise have been shown to result in a reduction in superoxide dismutase (SOD) levels, but did not alter either lipid or protein oxidation [25]. Increased cheese protein intake was shown to improve fasting insulin level in sarcopenic older persons [7]. The aetiology of sarcopenia is complex and multifaceted, involving homeostatic dysregulations in multiple physiological systems. They include imbalanced anabolic and catabolic metabolism that involves the actions of pancreatic, neuro-endocrine, muscle and adipocyte cytokine signaling, sex steroid anabolic homeostasis, hypothalamic–pituitary–adrenal (HPA) stress response, oxidative stress, and other mechanisms [30, 31]. The aim of this study was to explore the physiological responses associated with changes in muscle mass and function resulting from lifestyle intervention in frail older persons using a comprehensive range of blood biomarkers.

We previously reported a parallel group randomized controlled trial [32] multi-domain lifestyle interventions of six month duration (physical exercise, nutritional enrichment and cognitive training singly and in combination versus standard care control) among pre-frail and frail older persons living in the community. In that Frailty Intervention Trial (FIT) in Singapore, the effects of the different interventions on frailty outcomes (muscle strength, gait speed, body mass index, exhaustion and physical activity) at 3-month and 6-month has been reported [32]. Sarcopenia using DXA measure of appendicular lean muscle mass, muscle strength and functional performance was also assessed at baseline and follow up, and are the focus of examination of interventional outcome in this paper. We also collected archival baseline and follow-up blood specimens for analyses of known and potential blood biomarker indicators of molecular regulatory activity across multiple important physiological systems. In this study, we used these blood biomarkers to examine the physiological characteristics underlying the muscle mass and physical functional responses from lifestyle interventions in frail older adults.

## RESULTS

### Baseline characterization of sarcopenia and functional status

Participants in this randomized controlled trial (*n* = 242) were pre-frail or frail by the Fried physical phenotype criteria, of Chinese ethnicity with an average age of 70.0 years (SD: 4.7 years). Sarcopenia and non-sarcopenia elderly were comparable in demographic variables including mean age, proportions of gender and formal education level. Lower BMI, ASMI, lower limb strength and higher frailty score were observed in participants with sarcopenia compared to those without sarcopenia (*p* < 0.001). The sarcopenic participants also tend to show lower gait speed and physical activity, but the differences did not reach statistical significance. No difference was observed in participants with and without sarcopenia with regard to cognitive function (MMSE), mental health (GDS), and lung function (FEV1/FVC% predicted). There were similar proportions of sarcopenia participants who received different interventions. Table 1.

**Table 1:** Baseline characteristics and interventions by sarcopenia groups.

	**All subjects (*n* = 242)**	**Sarcopenic subgroups**			
		**Sarcopenia (*n* = 92)**	**Non-sarcopenia (*n* = 150)**	**t/χ^2^**	***p***
Age (years)	69.97 ± 4.70	69.95 ± 4.72	69.98 ± 4.71	–0.055	0.956
Gender (female)	150 (61.98)	59 (64.13)	91 (60.67)	0.290	0.590
Secondary and above education	75 (30.99)	33 (35.87)	42 (28.00)	1.651	0.199
BMI (kg/m^2^)	23.68 ± 3.47	21.31 ± 2.55	25.13 ± 3.15	–9.820	<0.001
ASMI (kg/m^2^)	6.11 ± 1.07	5.32 ± 0.82	6.60 ± 0.90	–11.092	<0.001
Lower limb strength (kg)	14.17 ± 5.01	12.27 ± 3.11	15.33 ± 5.57	–5.491	<0.001
Gait speed (second)	5.61 ± 1.65	5.76 ± 1.76	5.52 ± 1.57	1.074	0.284
Physical activity (min/day)	169.12 ± 12.63	158.20 ± 112.94	175.82 ± 112.29	–1.182	0.238
Frailty score	2.05 ± 0.85	2.28 ± 0.87	1.90 ± 0.80	3.494	<0.001
Interventions (*n* (%))					
Nutritional enrichment	47 (19.42)	16 (17.39)	31 (20.67)	6.187	0.186
Cognitive training	49 (20.25)	25 (27.17)	24 (16.00)		
Physical exercise	48 (19.83)	20 (21.74)	28 (18.67)		
Combined intervention	49 (20.25)	17 (18.48)	32 (21.33)		
Standard care	49 (20.25)	14 (15.22)	35 (23.30)		

Data are shown as mean ± SD or *n* (%). Abbreviations: BMI = body mass index; ASMI = Appendicular skeletal muscle index; MMSE = Mini-Mental State Examination; GDS = Geriatric Depression Scale.

### Multi-domain lifestyle interventions reversed sarcopenia

**Figure 1:**
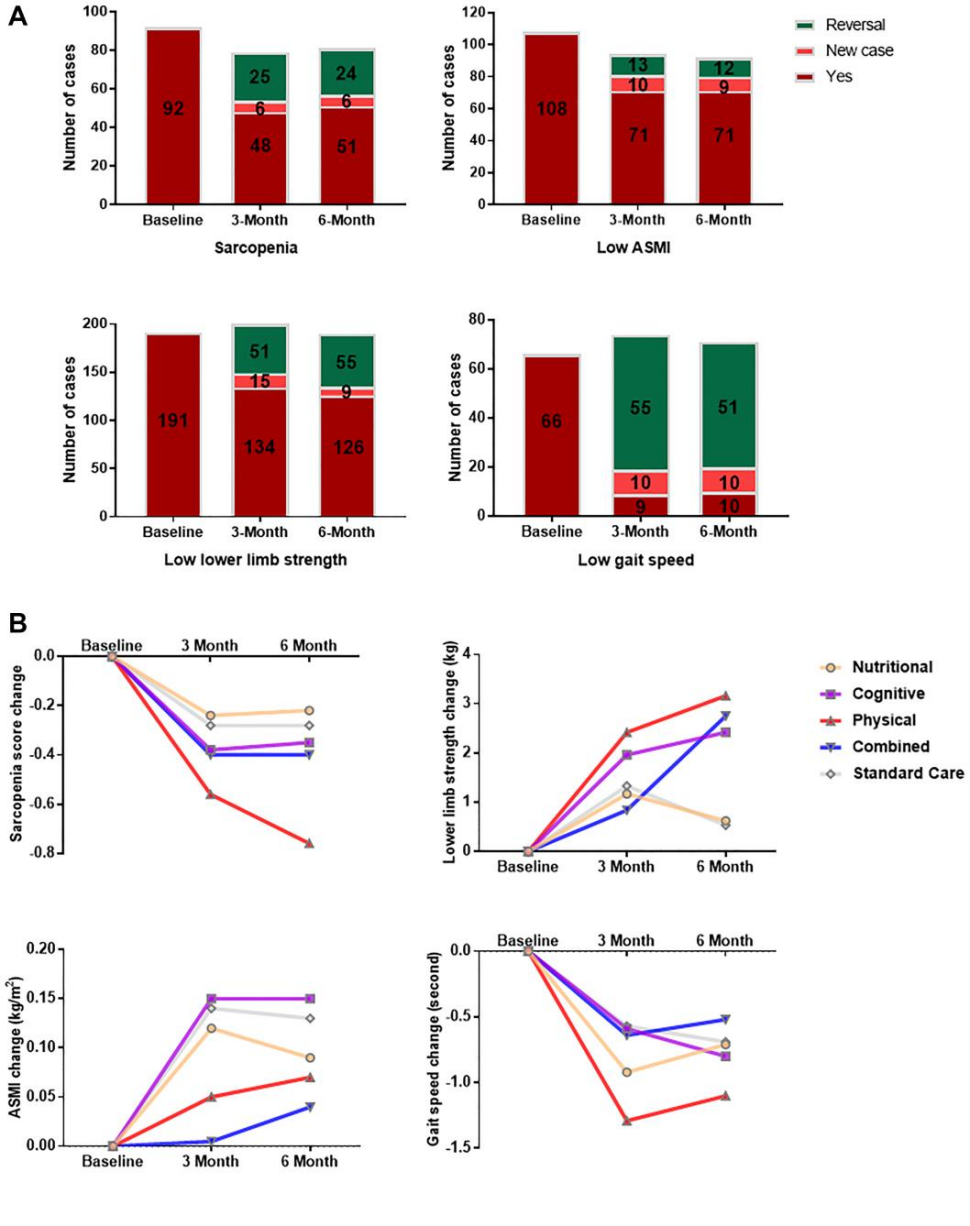
**Changes in sarcopenia and its components after multi-domain lifestyle intervention.** (**A**) Sarcopenia and component functional status among trial subjects (*n* = 242) at baseline, 3 month and 6 month. Reversal was defined as the presence at baseline and absence during follow-up. ASMI = Appendicular skeletal muscle index. (**B**) Changes in sarcopenia score and its components from baseline to 3-month and 6-month for each interventional arm. Sarcopenia score was calculated as the number of positive components for a participant. The components of sarcopenia included lower limb strength, ASMI, and gait speed. Participants were stratified by intervention groups consisting of nutritional enrichment (*n* = 47), cognitive training (*n* = 49), physical exercise (*n* = 48), combined intervention (*n* = 49), and standard care (*n* = 49) groups. ASMI = appendicular skeletal muscle index.

As shown in Figure 1A, overall there were 92 participants who had sarcopenia at baseline, and this figure was reduced to 54 and 57 respectively after 3 months and 6 months of intervention; 34.3% (25) and 32.0% (24) of the participants showed reversal of sarcopenia at 3-month and 6-month respectively. The proportions of reversal at 3-month and 6-month were highest for low gait speed, with 85.9% (55) and 83.6% (51) of the participants respectively, followed by low lower limb strength (27.6% (51) and 30.4% (55)) and low ASMI (15.5% (13) and 14.3% (12)).

Mixed model analysis (Table 2) indicated a significant main effect of time (*p* < 0.001). There was a significant decrease of sarcopenia score (*p* < 0.001) and significant increases of lower limb strength (*p* < 0.001), ASMI (*p* < 0.01 at 3-month and *p* < 0.001 at 6-month), and gait speed (*p* < 0.001) at 3-month and 6-month of intervention. There was a significant time × group interaction for the improvement of lower limb strength (*p* < 0.05) and a borderline time × group interaction for the reduction of sarcopenia score (*p* = 0.059). Improvements in sarcopenia (mean change = –0.76, *p* < 0.01 at 6-month) and gait speed (mean change = –1.29 second, *p* < 0.05 at 3-month) were most evident in the physical exercise group in comparison with the standard care group. Combined intervention (mean change = 2.75 kg, *p* < 0.05), physical exercise (mean change = 3.16 kg, *p* < 0.01), and cognitive training (mean change = 2.42 kg, *p* < 0.05) significantly enhanced lower limb strength at 6-month of intervention versus the standard care group. Figure 1B summarizes the changes in sarcopenia score and its components from baseline to 3-month and 6-month for each interventional arm.

**Table 2:** Effects of multi-domain lifestyle interventions on sarcopenia and components at 3 month and 6 month.

	**Lifestyle Interventions (mean ± SD)**					**Time**	**Group**	Time*Group
	**Nutritional (*n* = 47)**	**Cognitive (*n* = 49)**	**Physical (*n* = 48)**	**Combined (*n* = 49)**	**Standard Care (*n* = 49)**			
**Sarcopenia score**								
Baseline	1.47 ± 0.88	1.63 ± 0.86	1.75 ± 0.79	1.40 ± 0.79	1.33 ± 0.83	<0.001	0.307	0.059
3 Month^***^	1.11 ± 0.94	1.25 ± 0.87	1.12 ± 0.75	1.00 ± 0.72	1.02 ± 0.73			
6 Month^***^	1.14 ± 0.92	1.27 ± 0.75	0.93 ± 0.72	0.98 ± 0.88	1.02 ± 0.80			
**Change** (mean (95% CI))								
3 Month	–0.24 (–0.47, –0.02)	–0.38 (–0.65, –0.10)	–0.56 (–0.81, –0.32)	–0.40 (–0.62, –0.17)	–0.28 (–0.56, 0.01)			
6 Month	–0.22 (–0.45, 0.02)	–0.35 (–0.57, –0.13)	–0.76 (–0.99, –0.52)^§§^	–0.40 (–0.60, –0.19)	–0.28 (–0.54, –0.01)			
**ASMI, kg/m^2^**								
Baseline	6.14 ± 1.25	5.70 ± 0.93	6.12 ± 0.98	6.41 ± 1.15	6.21 ± 0.92	<0.001	0.066	0.635
3 Month^**^	6.28 ± 1.26	5.92 ± 1.06	6.21 ± 1.01	6.40 ± 1.14	6.21 ± 0.85			
6 Month^***^	6.26 ± 1.27	5.92 ± 1.09	6.24 ± 1.01	6.44 ± 1.14	6.19 ± 0.90			
**Change** (mean (95% CI))								
3 Month	0.12 (0.03, 0.20)	0.15 (–0.01, 0.30)	0.05 (–0.03, 0.12)	–0.01(–0.15, 0.13)	0.14 (0.03, 0.26)			
6 Month	0.09 (0.01, 0.18)	0.15 (–0.01, 0.30)	0.07 (–0.00, 0.15)	0.04 (–0.05, 0.12)	0.13 (0.01, 0.25)			
**Lower limb strength, kg**								
Baseline	14.25 ± 5.86	12.71 ± 3.40	13.72 ± 4.17	15.10 ± 6.15	15.06 ± 4.74	<0.001	0.255	0.012
3 Month^***^	15.43 ± 5.88	14.72 ± 5.77	16.13 ± 5.54	16.02 ± 6.17	16.22 ± 5.54			
6 Month^***^	15.05 ± 4.79	15.16 ± 5.24	16.88 ± 5.47	17.77 ± 6.79	15.17 ± 4.47			
**Change** (mean (95% CI))								
3 Month	1.17 (–0.21, 2.54)	1.96 (0.37, 3.56)	2.42 (1.05, 3.78)	0.84 (–0.47, 2.15)	1.33 (–0.38, 3.04)			
6 Month	0.62 (–0.59, 1.83)	2.42 (1.04, 3.80)^§^	3.16 (1.71, 4.61)^§§^	2.75 (1.46, 4.05)^§^	0.53 (–0.88, 1.95)			
**Gait speed, second**								
Baseline	5.75 ± 1.75	5.39 ± 1.17	6.08 ± 2.08	5.39 ± 1.25	5.47 ± 1.77	<0.001	0.382	0.355
3 Month^***^	4.83 ± 1.20	4.76 ± 0.98	4.79 ± 0.89	4.74 ± 1.20	4.90 ± 1.73			
6 Month^***^	4.99 ± 1.03	4.62 ± 0.81	4.97 ± 1.04	4.83 ± 1.13	4.79 ± 0.99			
**Change** (mean (95% CI))								
3 Month	–0.92 (–1.43, –0.41)	–0.59 (–0.98, –0.20)	–1.29 (–1.87, –0.71)^§^	–0.64 (–1.06, –0.23)	–0.57 (–1.09, –0.04)			
6 Month	–0.71 (–1.22, –0.21)	–0.80 (–1.16, –0.43)	–1.10 (–1.73, –0.48)	–0.52 (–0.92, –0.11)	–0.69 (–1.19, –0.18)			

Abbreviations: ASMI = Appendicular skeletal muscle index. ^**^p < 0.01, ^***^p < 0.001 vs. baseline level; ^§^*p* < 0.05, ^§§^*p* < 0.01 vs. standard care group.

### Responsiveness to interventions of sarcopenia versus non-sarcopenia participants

Although sarcopenic elderly were slower and more inactive at baseline, results (Table 3) showed that they were as responsive as their non-sarcopenia counterparts to the interventions. Both groups had overall significant improvements in lower limb strength (*p* < 0.001), ASMI (*p* < 0.01 for sarcopenia and *p* < 0.05 for non-sarcopenia), and gait speed (*p* < 0.001) at 3-month and 6-month of intervention. No difference between sarcopenia and non-sarcopenia groups was observed in the change of sarcopenia components at 3-month and 6-month, except that sarcopenia elderly had marginally more increase in 6-month ASMI than those without sarcopenia (*p* = 0.051). The comparable responsiveness of sarcopenia versus non-sarcopenia participants overall was replicated in the detailed data in each interventional arm shown in Table 4.

**Table 3:** Physical function improvements at 3-month and 6-month interventions in sarcopenia versus non-sarcopenia elderly.

	**All subjects (*n* = 242)**	**Sarcopenic subgroups**			
		**Sarcopenia (*n* = 92)**	**Non-Sarcopenia (*n* = 150)**	***t***	***p***
Lower limb strength, kg					
Baseline (0M)	14.17 ± 5.01	12.27 ± 3.11	15.33 ± 5.57	–5.491	<0.001
3 Month (3M)	15.71 ± 5.76^***^	13.93 ± 4.13^***^	16.75 ± 6.31^**^	–4.132	<0.001
6 Month (6M)	16.04 ± 5.50^***^	14.08 ± 4.71^***^	17.17 ± 5.61^***^	–4.256	<0.001
Change (3M-0M)%	15.57 ± 38.15	17.47 ± 36.55	14.47 ± 39.12	0.580	
Change (6M-0M)%	18.28 ± 36.22	19.05 ± 38.46	17.84 ± 34.99	0.243	
F, *p*	19.612, <0.001	10.160, <0.001	11.343, <0.001		
ASMI, kg/m^2^					
Baseline (0M)	6.11 ± 1.07	5.32 ± 0.82	6.60 ± 0.90	–11.092	<0.001
3 Month (3M)	6.21 ± 1.07^**^	5.37 ± 0.74	6.66 ± 0.95^*^	–10.603	<0.001
6 Month (6M)	6.21 ± 1.09^***^	5.35 ± 0.76^**^	6.68 ± 0.95	–10.802	<0.001
Change (3M-0M)%	1.65 ± 6.18	2.20 ± 6.84	1.35 ± 5.80	0.928	
Change (6M-0M)%	1.69 ± 5.82	1.73 ± 7.04	1.68 ± 5.06	0.051	
F, *p*	8.104, <0.001	6.486, 0.002	3.513, 0.035		
Gait speed, second					
Baseline (0M)	5.61 ± 1.65	5.76 ± 1.76	5.52 ± 1.57	1.074	0.284
3 Month (3M)	4.84 ± 1.23^***^	4.72 ± 1.01^***^	4.85 ± 1.33^***^	–0.834	0.405
6 Month (6M)	4.84 ± 1.01^***^	4.92 ± 1.02^***^	4.79 ± 1.00^***^	0.942	0.347
Change (3M-0M)%	–10.43 ± 24.17	–13.29 ± 23.56	–8.79 ± 24.44	–1.378	
Change (6M-0M)%	–9.04 ± 24.45	–9.37 ± 26.11	–8.85 ± 23.53	–0.155	
F, *p*	28.155, <0.001	13.688, <0.001	16.408, <0.001		

Abbreviations: ASMI = Appendicular skeletal muscle index. ^*^p < 0.05, ^**^p < 0.01, ^***^p < 0.001 vs. baseline level.

**Table 4:** Physical function changes of sarcopenia and non-sarcopenia elderly for each intervention arm.

	**Sarcopenia**	**Non-Sarcopenia**	***t***	***p***	**Sarcopenia**	**Non-Sarcopenia**	***t***	***p***
	**Nutritional Enrichment**				**Physical Exercise**			
	*n* = 16	*n* = 31			*n* = 25	*n* = 24		
Lower limb strength, kg								
Baseline	11.02 ± 2.37	15.91 ± 6.44	–3.765	<0.001	11.97 ± 3.00	13.47 ± 3.67	–1.566	0.124
6 Month	12.53 ± 3.58	16.40 ± 4.87	–2.708	0.010	13.52 ± 4.05	16.60 ± 5.80	–2.032	0.048
* t*, *p*	–2.154, 0.049	–0.073, 0.942			–1.828, 0.083	–3.069, 0.005		
ASMI,kg/m^2^								
Baseline	4.87 ± 0.66	6.79 ± 0.94	–7.247	<0.001	5.20 ± 0.79	6.23 ± 0.78	–4.559	<0.001
6 Month	4.77 ± 0.53	6.89 ± 0.91	–8.898	<0.001	5.36 ± 0.74	6.43 ± 1.12	–3.511	0.001
* t*, *p*	–0.383, 0.710	–2.404, 0.024			–1.868, 0.078	–1.401, 0.177		
Gait speed, second								
Baseline	6.39 ± 2.58	5.41 ± 1.03	1.457	0.163	5.59 ± 1.00	5.18 ± 1.32	1.214	0.231
6 Month	5.53 ± 1.17	4.70 ± 0.83	2.690	0.010	4.87 ± 0.90	4.40 ± 0.66	1.988	0.053
* t*, *p*	1.482, 0.160	2.935, 0.007			3.642, 0.002	2.785, 0.011		
	**Cognitive Training**				**Combined Intervention**			
	*n* = 20	*n* = 28			*n* = 17	*n* = 32		
Lower limb strength, kg								
Baseline	13.07 ± 2.79	14.18 ± 4.92	–0.995	0.325	12.53 ± 3.99	16.46 ± 6.69	–2.214	0.032
6 Month	16.58 ± 6.26	17.09 ± 4.94	–0.315	0.754	13.80 ± 3.91	19.63 ± 7.10	–2.961	0.005
* t*, *p*	–2.818, 0.011	–3.339, 0.002			–1.873, 0.082	–3.865, <0.001		
ASMI, kg/m^2^								
Baseline	5.53 ± 0.80	6.54 ± 0.88	–4.059	<0.001	5.36 ± 0.71	6.98 ± 0.93	–6.266	<0.001
6 Month	5.43 ± 0.75	6.66 ± 0.87	–4.514	<0.001	5.44 ± 0.79	6.95 ± 0.93	–5.346	<0.001
* t*, *p*	–0.600, 0.559	–2.239, 0.034			–2.065, 0.058	0.341, 0.736		
Gait speed, second								
Baseline	5.76 ± 2.07	6.30 ± 2.09	–0.894	0.376	5.33 ± 1.31	5.42 ± 1.24	–0.239	0.812
6 Month	4.84 ± 0.99	5.07 ± 1.08	–0.733	0.467	4.79 ± 0.99	4.85 ± 1.21	–0.176	0.861
* t*, *p*	1.786, 0.090	3.145, 0.004			1.265, 0.226	2.188, 0.036		
	**Standard Care**				**Active Intervention**			
	*n* = 14	*n* = 35			*n* = 78	*n* = 115		
Lower limb strength, kg								
Baseline	12.74 ± 3.22	15.98 ± 4.96	–2.698	0.010	12.18 ± 3.11	15.13 ± 5.74	–4.609	<0.001
6 Month	13.23 ± 4.07	15.94 ± 4.45	–1.900	0.064	14.24 ± 4.83	17.54 ± 5.88	–3.960	<0.001
* t*, *p*	–0.446, 0.664	–0.610, 0.546			–4.328, <0.001	–5.166, <0.001		
ASMI, kg/m^2^								
Baseline	5.69 ± 0.97	6.41 ± 0.83	–2.602	0.012	5.25 ± 0.77	6.66 ± 0.92	–11.452	<0.001
6 Month	5.68 ± 0.79	6.41 ± 0.87	–2.493	0.017	5.29 ± 0.75	6.75 ± 0.96	–10.806	<0.001
* t*, *p*	–0.151, 0.882	–3.877, <0.001			–2.513, 0.015	–2.465, 0.015		
Gait speed, second								
Baseline	5.85 ± 1.74	5.32 ± 1.78	0.945	0.349	5.74 ± 1.78	5.58 ± 1.50	0.656	0.513
6 Month	4.59 ± 0.99	4.86 ± 1.00	–0.836	0.407	4.98 ± 1.02	4.77 ± 1.00	1.382	0.169
* t*, *p*	2.214, 0.047	1.770, 0.086			3.657, <0.001	5.385, <0.001		

Abbreviations: ASMI = Appendicular skeletal muscle index.

### Baseline characteristics of subjects with post-interventional sarcopenia reversal

**Figure 2:**
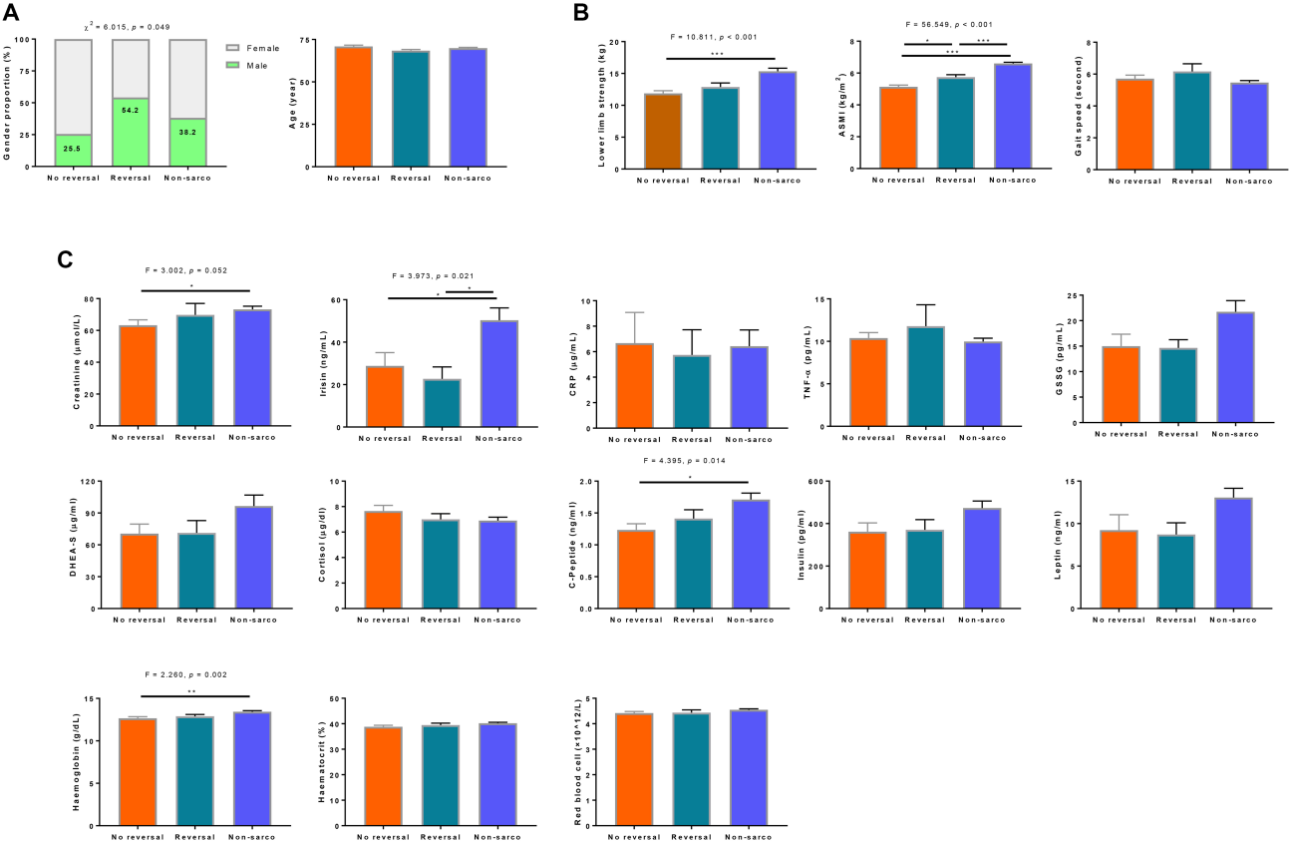
**Characterization of demographics.** (**A**) sarcopenia components, (**B**) and related biomarker, (**C**) features of elderly with sarcopenia reversal at the end of the intervention. No reversal was defined as the presence of sarcopenia at both baseline and 6-month (*n* = 51). Reversal group included elderly who had the presence of sarcopenia at baseline and absence of sarcopenia at 6-month (*n* = 24). Non-sarcopenia was classified as the absence of sarcopenia at both baseline and 6-month (*n* = 131). CRP = C-reactive protein, TNF-α = tumor necrosis factor alpha, DHEA-S = dehydroepiandrosterone sulfate.

The features of sarcopenia reversal were explored by comparing the demographics, physical and related blood biomarkers in the groups with sarcopenia reversal (presence of sarcopenia at baseline and absence of sarcopenia at 6-month, *n* = 24) versus the sarcopenia non-reversal group (presence of sarcopenia at both baseline and 6-month, *n* = 51), and the non-sarcopenia group (absence of sarcopenia at both baseline and 6-month, *n* = 131) groups. There was a much higher proportion of male participants in the reversal group (54.2%) than in the sarcopenia non-reversal (25.5%) and the non-sarcopenia (38.2%) groups (*p* < 0.05). Figure 2A. Participants whose sarcopenia were reversed at 6-month had significantly higher baseline ASMI levels than those who remained sarcopenic at 6-month (*p* < 0.05). There was no significant difference in baseline lower limb strength between the sarcopenia reversal and non-sarcopenia groups (*p* > 0.05), but the sarcopenia non-reversal group had significantly lower baseline lower limb strength than those in the non-sarcopenia group (*p* < 0.001). No difference was observed for gait speed among the three groups (*p* > 0.05). Figure 2B.

### Blood biomarkers of physiological functions

#### Cross-sectional differences by sarcopenia groups

At baseline, sarcopenic participants showed lower levels of circulating biomarker indicators of multiple physiological functions compared to non-sarcopenic participants, for those that were particularly related to muscle metabolism (creatinine: *p* < 0.05; irisin: *p* < 0.01), oxidative stress regulation (GSSG: *p* < 0.01), sex hormone and HPA stress (DHEA-S: *p* < 0.05), insulin signaling (c-peptide: *p* < 0.01; insulin: *p* < 0.05; leptin: *p* < 0.05), and tissue oxygenation (haemoglobin: *p* < 0.001; haematocrit: *p* < 0.05; red blood cell: *p* < 0.05). Table 5. The linear regression models also revealed the associations of these biomarkers with component measures of lower limb strength, ASMI and gait speed. Supplementary Table 1.

**Table 5:** Circulating biomarker levels in sarcopenia and non-sarcopenia subgroups at pre and post interventions.

		**All subjects**	**Sarcopenia Subgroups**				**Pairwise comparison (6 month vs baseline)**		
			**Sarcopenia**	**Non-Sarcopenia**					
		***N* = 242**	***N* = 92**	***N* = 150**	***t***	***p***	**Mean**	***t***	***p***
**Baseline**									
Muscle metabolism	Creatinine (μmol/L)	70.11 ± 22.72	65.46 ± 24.39	72.85 ± 21.29	–2.347	0.020			
	Irisin (ng/mL)	38.04 ± 47.08	24.99 ± 31.40	45.94 ± 52.84	–3.130	0.002			
Inflammation and	CRP (μg/mL)	6.17 ± 13.47	6.32 ± 13.78	6.08 ± 13.34	0.128	0.898			
Anti-oxidation	TNF-α (pg/mL)	10.24 ± 5.05	10.69 ± 6.31	9.99 ± 4.17	0.977	0.329			
	GSSG (pg/mL)	18.55 ± 17.39	14.47 ± 11.36	21.02 ± 19.84	–2.647	0.009			
Steroid hormone	DHEA-S (μg/ml)	86.93 ± 86.32	69.62 ± 63.42	97.22 ± 99.75	–2.339	0.021			
regulation	Cortisol (μg/dl)	6.99 ± 2.59	7.29 ± 2.60	6.81 ± 2.57	1.180	0.240			
Insulin signaling	C-peptide (ng/ml)	1.54 ± 0.85	1.32 ± 0.59	1.67 ± 0.95	–2.981	0.003			
	Insulin (pg/ml)	435.44 ± 282.99	375.15 ± 232.77	472.32 ± 304.05	–2.171	0.031			
	Leptin (ng/ml)	11.13 ± 10.09	8.82 ± 9.30	12.54 ± 10.33	–2.339	0.021			
Tissue oxygenation	Haemoglobin (g/dL)	13.26 ± 1.35	12.84 ± 1.18	13.51 ± 1.38	–3.651	<0.001			
	Haematocrit (%)	40.03 ± 3.61	39.30 ± 3.29	40.47 ± 3.73	–2.116	0.036			
	Redbloodcell (×10^12^/L)	4.52 ± 0.38	4.44 ± 0.37	4.57 ± 0.39	–2.115	0.036			
**6 Month**									
Muscle metabolism	Creatinine (μmol/L)	69.91 ± 23.34	67.94 ± 28.73	71.03 ± 19.73	–0.759	0.450	–0.327	–0.560	0.576
	Irisin (ng/mL)	36.08 ± 42.16	30.17 ± 42.98	39.38 ± 41.57	–1.264	0.208	–2.361	–0.795	0.428
Inflammation and	CRP (μg/mL)	2.97 ± 3.81	2.69 ± 2.60	3.13 ± 4.36	–0.707	0.480	–3.259	–3.365	<0.001
Anti-oxidation	TNF-α (pg/mL)	9.52 ± 3.11	9.35 ± 3.42	9.62 ± 2.93	0.359	0.589	–0.915	–3.031	0.003
	GSSG (pg/mL)	18.32 ± 18.29	13.33 ± 7.11	21.01 ± 21.65	–3.054	0.003	–0.807	–0.626	0.532
Steroid hormone	DHEA-S (μg/ml)	84.51 ± 76.79	74.02 ± 66.55	90.49 ± 81.80	–1.248	0.214	–0.528	–0.124	0.902
Regulation	Cortisol (μg/dl)	7.28 ± 2.51	7.84 ± 2.57	6.96 ± 2.43	2.047	0.043	0.143	0.555	0.580
Insulin signaling	C-peptide (ng/ml)	1.49 ± 0.72	1.34 ± 0.61	1.57 ± 0.77	–1.879	0.062	–0.090	–1.377	0.171
	Insulin (pg/ml)	414.79 ± 269.35	375.10 ± 226.08	437.91 ± 290.31	–1.353	0.178	–37.912	–1.769	0.079
	Leptin (ng/ml)	11.70 ± 12.71	9.97 ± 12.44	12.68 ± 12.82	–1.247	0.214	–0.180	–0.252	0.802
Tissue oxygenation	Haemoglobin (g/dL)	13.31 ± 1.36	12.92 ± 1.23	13.53 ± 1.39	–2.907	0.004	0.075	1.321	0.188
	Haematocrit (%)	40.52 ± 3.77	39.40 ± 3.53	41.21 ± 3.78	–2.666	0.009	0.123	0.710	0.480
	Redbloodcell (×10^12^/L)	4.55 ± 0.35	4.44 ± 0.33	4.60 ± 0.35	–2.323	0.022	–0.012	–0.698	0.487

Abbreviations: CRP = C - reactive protein, TNF-α = tumor necrosis factor alpha, DHEA-S = dehydroepiandrosterone sulfate.

#### Differences in pre- and post-interventional changes by sarcopenia groups

The differences in the levels of these biomarkers between the sarcopenia and non-sarcopenia groups that were observed at baseline pre-intervention were mostly reduced after 6 months of intervention, resulting in comparable levels of biomarkers of muscle metabolism (creatinine; irisin) and insulin signaling (c-peptide; insulin; leptin). There remained significant differences in the biomarker levels of anti-oxidation (GSSG) and tissue oxygenation (haemoglobin; haematocrit; red blood cell) between the two groups. With regard to sex hormone and HPA stress, the difference in DHEA-S levels disappeared while the difference in cortisol levels emerged at 6 months between sarcopenia and non-sarcopenia elderly. Table 5.

With regard to inflammatory biomarkers, no difference between sarcopenia and non-sarcopenia groups were observed at baseline or 6 months of intervention. There were substantial reductions in the levels of inflammation indicators such as CRP (from 6.17 μg/mL to 2.97 μg/mL) and TNF-α (from 10.24 pg/mL to 9.52 pg/mL), and this was especially pronounced in the sarcopenia group (CRP: from 6.32 μg/mL to 2.69 μg/mL; TNF-α: from 10.69 pg/mL to 9.35 pg/mL).

#### Differences by specific intervention domains

The level of TNF-α was most significantly reduced by combined intervention (*p* < 0.05) and the level of CRP was most significantly reduced by cognitive training (*p* < 0.05). Creatinine level decreased significantly in the standard care group (*p* <0.001), but was preserved in the physical exercise, combined intervention and nutritional enrichment groups (*p* > 0.05), or increased in the cognitive training group (*p* < 0.05). All active interventions also preserved c-peptide and insulin levels whereas they were significantly decreased in the standard care group (*p* < 0.05 for c-peptide and *p* < 0.01 for insulin). Supplementary Table 2.

#### Differences by sarcopenia reversal groups

We compared the baseline levels of blood biomarkers among non-sarcopenia, sarcopenia non-reversal and sarcopenia reversal groups. As shown in Figure 2C, sarcopenia reversal tended to be predicted by higher baseline creatinine, c-peptide, and haemoglobin levels.

## DISCUSSION

In this secondary analysis of data from our previous studies, we observed that multi-domain physical, nutritional and cognitive interventions among pre-frail and frail older adults were associated with favorable changes in sarcopenia and blood biomarkers underlying the muscle mass and physical functional response to intervention. As previously reported, the data are highly consistent with previous studies in showing that physical exercise alone or in combination with cognitive and nutritional intervention was most efficacious in improving muscle mass, lower limb strength and gait speed. The physical exercise in this study was of moderate and gradually increasing intensity and well tolerated with high adherence rate (85%). Perhaps unsurprisingly, there was limited effect observed with nutritional intervention delivered with a traditional oral nutrition supplement and not with a formulation with high content of leucine or whey protein or vitamin D, which have been shown in more recent studies to increase muscle mass and muscle function in sarcopenic and malnourished older patients [8–11]. The observed effects of cognitive training on enhancing gait speed is expected given the important role of cognitive processing especially executive functioning in balance and gait and functional mobility. The concurrent improvement in lower limb strength was somewhat surprising, and has not been reported by other interventional studies that only assessed gait and balance but not muscle strength. The mechanistic relationships are still not fully understood, although age-related loss of motor neuron can reduce muscle mass and strength, and sarcopenia is believed to be an important link between the comorbidity of cognitive impairment and physical functional impairment [28, 33]. We also speculate that the observed effect may be attributed to the extraordinary physical activity associated with participation in cognitive training classes in habitually sedentary frail individuals.

Chronic low-grade inflammation associated with oxidative stress is believed to be a major underlying mechanism of aging and aging-related diseases including sarcopenia [34]. Inflammatory markers such as CRP and IL-6 are reported to be associated with decreased muscle mass and strength [35, 36], and the reduction of inflammation is believed to directly or indirectly ameliorate age-related muscle loss [37]. In the present study, inflammatory levels are observed to be reduced especially by combined intervention, as evidenced by the significant drops in CRP and TNF-α levels. However, the levels of these inflammatory markers were not associated with sarcopenia status or reduction. Thus, the reduction of inflammation may not be the primary underlying mechanism of the response of sarcopenic elderly to lifestyle interventions. On the other hand, sarcopenic elderly showed lower levels of the powerful antioxidant, GSSG. Aging may predispose skeletal muscles to increased oxidative stress at rest and during disuse atrophy, leading to disuse- and sarcopenia-associated muscle loss. Previous studies have also reported the independent association of low circulating level of another antioxidant, carotenoids, with the decrease of skeletal muscle strength [38] and development of walking disability [39]. A randomized controlled trial has also shown that soy protein supplementation and exercise result in a reduction in superoxide dismutase (SOD) levels, but did not alter either lipid or protein oxidation [25].

Beyond inflammation and oxidative stress, we found that circulating biomarkers related to other physiological functions involved in the causative cascade of sarcopenia including muscle metabolism (creatinine, irisin), steroid hormone regulation (DHEA-S, cortisol), insulin signaling (c-peptide, insulin, leptin), and tissue oxygenation (haemoglobin, haematocrit, red blood cell) are linked to sarcopenia baseline status and sarcopenia reduction post-intervention. The association appears to be prominent for creatinine, c-peptide, and haemoglobin levels. Serum creatinine correlates well with dual-energy X-ray absorptiometry (DXA)-measured lean body mass and is regarded as a surrogate of muscle mass and a biomarker for sarcopenia [40, 41]. The observation that serum creatinine predicts sarcopenia reduction in this study suggests its possible prognostic value in interventional studies of sarcopenia.

Sufficient insulin secretion and efficient insulin signaling play a critical role in promoting protein synthesis, maintaining muscle function, and preventing muscle mass loss and sarcopenia [42, 43]. Consonantly in this study, both insulin and c-peptide levels were associated with sarcopenia status, ASMI, and lower limb strength, and were preserved by any of the active interventions but decreased in the standard care group. Furthermore, c-peptide was found to predict sarcopenia reduction. The levels of c-peptide are not affected by insulin injections or liver metabolization and are thus considered a better measure of portal insulin secretion than insulin itself [44].

Our observation of the association between haemoglobin and sarcopenia status and reduction, ASMI, muscle strength, and gait speed is consistent with reports of the association of haemoglobin and anemia with sarcopenia [45, 46]. Low haemoglobin concentration may impair tissue delivery of oxygen, creating sub-clinical hypoxia in skeletal muscle that in the long run impairs muscle strength and performance. Taken together, these circulating biomarkers associated with sarcopenia status and reduction may serve as easily accessible and cost-effective means of measuring physiological responses to interventions aimed at reducing sarcopenia in clinical research and practice.

Our analyses suggest that the effects of lifestyle interventions in reversing sarcopenia in community-dwelling older persons are accompanied by multi-level targeted physiological changes that include age-related inflammation and the anabolic and catabolic mechanisms of regulating hormones and tissue oxygenation involved in the causation of sarcopenia. Nevertheless, as the study was designed for frailty as the primary outcome among participants with pre-frailty and frailty, sarcopenia was not the primary readout. The findings obtained in this secondary analysis of data should therefore be validated in an independent dataset with sarcopenia as the primary outcome. The suggestive results of sarcopenia reduction based on multiple subgroup analyses are indirect observations of the effects of interventions, and should therefore be viewed with some circumspection. The exploratory nature of the study is emphasized, as we measured a wide array of biomarkers and evaluated all observable changes in physiological biomarkers associated with the interventions. Spurious significant results are possible, but some meaningful changes that stood out were highly consistent with previous reports in the literature.

## MATERIALS AND METHODS

### Study design and participants

The parallel group randomized controlled trial of the Singapore Frailty Intervention Trial (FIT, http://clinicaltrials.gov/ identifier NCT00973258) has been described in a previous publication [32], and is briefly summarized here. The eligible study subjects were 246 community-dwelling older persons with the physical frailty phenotype of pre-frailty or frailty from Fried at al. [47]. These older persons were randomized to receive one of 5 interventions of 24-week duration each: physical exercise, nutritional enrichment, cognitive training, combined intervention, or standard care. The study was approved by National Health Group Domain Specific Review Board (DSRB) of Singapore, and all participants provided written informed consent.

### Sarcopenia measurement

Sarcopenia, which is the primary outcome of this study, was determined based on appendicular lean mass, lower limb strength and gait speed, according to the Asian Working Group for Sarcopenia (AWGS) criteria [48] released in 2014. Appendicular lean body mass was measured by DXA scan with the Hologic^®^ densitometer. Four participants refused to undergo the DXA scan and were removed from the analysis. A participant was categorized as having sarcopenia if he/she had both low appendicular skeletal muscle index (ASMI) and low knee extension strength (KES, less than or equal to 18 kg for men and less than or equal to 16 kg for women) and/or gait speed (GS, less than or equal to 0.8 m/s). Sarcopenia score was calculated as the number of positive components. KES was measured using the strap and strain gauge assembly component of the Physiological Profile Assessment and GS was assessed by the 6-meter fast gait speed test.

### Multi-domain lifestyle interventions

Details of the interventions have been described in a previous publication [32] and are briefly summarized below:

#### Physical exercise

Participants performed resistance exercise integrated with functional tasks and balance training exercise involving functional strength, sensory input, and added attentional demands. Each program started with two sessions per week (90-minute each session) for 12 weeks in classes supervised by a qualified trainer, followed by home-based exercise for another 12 weeks. The exercise was tailored to the functional needs of the participants, maintaining a moderate intensity that focuses on long-term sustainability and enjoyment, with balance and strength as the key components.

#### Nutritional enrichment

Participants were provided nutritional advice and supervision with additional daily supplements of a commercial formula (Fortisip Multi Fibre, Nutricia: 200 mL liquid formula with 300 kcal carbohydrate (49%), fat (35%), protein (35%), and dietary fiber (4.6 g)), a calcium and vitamin D supplement tablet (Caltrate; 600 mg calcium and 200 IU vitamin D), an iron and folate capsule (Sangobion, Merck; 1 mg folate and 29 mg iron), and a vitamin B6 and B12 tablet (Neuroforte; 200 mg B6 and 200 μg B12) for 24 weeks. This regime was designed to increase caloric intake by 20% and provide additional 1/3 of the RADs of vitamins and minerals.

#### Cognitive training

Cognitive enhancing activities included learning strategies for recalling verbal and visual information, tasks such as “spot the differences”, categorical naming, coding to enhance attention and processing speed, and matrix reasoning exercises, mazes and tangram-like games aimed at enhancing reasoning and problem-solving abilities. Participants attended intensive cognitive training classes for the first 12 weeks followed by 12-week fortnightly 2-hour “booster” sessions where they reviewed the cognitive skills learned in the intensive classes.

#### Combined intervention

Elderly in this group underwent all three aforementioned interventions.

#### Standard care

Participants had access to standard community-based social, recreational and day care rehabilitation services and were given an equal volume of artificially sweetened, vanilla-flavored liquid (ingredients: non-dairy creamer, liquid caramel, sugar and water), two capsules, and one tablet (ingredients: corn-starch, lactose and magnesium stearate) that were identical in appearance to the active nutritional supplements. Both the active supplement and standard care placebo were administered by interventional nurses who had no knowledge of the participants’ group allocation.

### Pre- and post-intervention assessments

At baseline pre-intervention and 3 months and 6 months post-intervention, participants underwent interviews and testing that included DXA scan, lower limb strength measurement, and 6-meter fast gait speed test for the assessment and diagnosis of sarcopenia. Blood was drawn after over-night fasting, and serum was isolated and stored in –80°C freezer until measurements.

#### Blood biomarkers

Archival serum specimens were thawed, and quantitative assays were performed for analytes of hormones, cytokines, metabolites, and other biomarkers that were identified from literature search to be involved in homeostatic processes associated with sarcopenia. Levels of CRP, cortisol, dehydroepiandrosterone sulfate (DHEA-S) and irisin were measured via the enzyme-linked immunosorbent assay (ELISA). Insulin, leptin and C-peptide levels were quantified using the Luminex assay (Merck Millipore). Measurements of creatinine, TNF-α, haemoglobin, haematocrit and red blood cell count were performed using standard clinical chemistry methods at NUH Referral Laboratories.

### Statistical analysis

The efficacy of interventions was examined using intention-to-treat (ITT) analysis. Differences in sarcopenia versus non-sarcopenia groups were compared by independent *t*-test for continuous variables and chi-square test for categorical variables. The linear mixed model was applied to investigate the effects of treatment group, time and group × time interaction as fixed factors. For variables with significant group × time interaction indicating changing group effect over time, the simple main effect of treatment group was further evaluated at each time point using one-way analysis of variance (ANOVA) with Bonferroni post-hoc adjustments. All data analyses were performed using IBM SPSS 21 software (IBM, USA). Figures were generated using GraphPad Prism 7.

## Supplementary Material

Supplementary Tables
