# Supplementary material for: Effects of multi-domain lifestyle interventions on sarcopenia measures and blood biomarkers: secondary analysis of a randomized controlled trial of community-dwelling pre-frail and frail older adults
Source: Aging (Albany NY). 2021 Mar 19;13(7):9330–47. doi: 10.18632/aging.202705 (PMC8064206; doi:10.18632/aging.202705)
Supplement: Supplementary Tables [file aging-13-202705-s001.pdf]

SUPPLEMENTARY TABLES

Supplementary Table 1. Relations of baseline measures of circulating biomarkers to sarcopenia score and its components.

	Sarcopenia score			Lower limb strength, kg		
	**B ± SE**	**β**	***p***	**B ± SE**	**β**	***p***
Creatinine (μmol/L)	–0.005 ± 0.002	–0.146	0.031	0.083 ± 0.014	0.366	<0.001
Irisin (ng/mL)	–0.001 ± 0.001	–0.053	0.499	–0.016 ± 0.009	–0.141	0.072
CRP (μg/mL)	0.001 ± 0.004	0.016	0.818	0.050 ± 0.025	0.134	0.047
TNF-α (pg/mL)	0.006 ± 0.011	0.036	0.599	0.102 ± 0.068	0.101	0.136
GSSG (pg/mL)	–0.004 ± 0.004	–0.084	0.295	–0.019 ± 0.024	–0.064	0.424
DHEA-S (μg/ml)	–0.002 ± 0.001	–0.163	0.034	0.003 ± 0.005	0.053	0.493
Cortisol (μg/dl)	0.020 ± 0.025	0.061	0.434	0.094 ± 0.159	0.046	0.555
C-peptide (ng/ml)	–0.185 ± 0.074	–0.190	0.014	1.181 ± 0.480	0.188	0.015
Insulin (pg/ml)	–0.001 ± 0.000	–0.174	0.025	0.003 ± 0.001	0.139	0.075
Leptin (ng/ml)	–0.003 ± 0.006	–0.031	0.688	–0.066 ± 0.041	–0.123	0.112
Haemoglobin (g/dL)	–0.182 ± 0.040	–0.299	<0.001	1.207 ± 0.241	0.324	<0.001
Haematocrit (%)	–0.058 ± 0.017	–0.254	<0.001	0.620 ± 0.099	0.429	<0.001
Red blood cell ($\times 10^{12}$/L)	–0.420 ± 0.163	–0.196	0.011	5.053 ± 0.992	0.367	<0.001
	**ASMI, kg/m²**			**Gait speed, second**		
	**B ± SE**	**β**	***p***	**B ± SE**	**β**	***p***
Creatinine (μmol/L)	0.020 ± 0.003	0.423	<0.001	0.000 ± 0.005	0.005	0.942
Irisin (ng/mL)	–0.001 ± 0.002	–0.033	0.679	–0.000 ± 0.003	0.000	0.999
CRP (μg/mL)	–0.005 ± 0.006	–0.060	0.374	–0.003 ± 0.008	–0.025	0.712
TNF-α (pg/mL)	0.004 ± 0.014	0.018	0.788	0.021 ± 0.022	0.066	0.332
GSSG (pg/mL)	0.014 ± 0.005	0.227	0.004	–0.003 ± 0.008	–0.027	0.737
DHEA-S (μg/ml)	0.002 ± 0.001	0.173	0.025	–0.003 ± 0.002	–0.123	0.110
Cortisol (μg/dl)	0.001 ± 0.033	0.003	0.966	0.022 ± 0.054	0.032	0.680
C-peptide (ng/ml)	0.227 ± 0.098	0.177	0.022	–0.063 ± 0.163	–0.030	0.702
Insulin (pg/ml)	0.001 ± 0.000	0.157	0.044	0.000 ± 0.000	–0.028	0.724
Leptin (ng/ml)	–0.006 ± 0.008	–0.056	0.477	0.004 ± 0.014	0.023	0.765
Haemoglobin (g/dL)	0.452 ± 0.045	0.566	<0.001	–0.166 ± 0.084	–0.134	0.049
Haematocrit (%)	0.176 ± 0.019	0.576	<0.001	–0.057 ± 0.037	–0.117	0.125
Red blood cell ($\times 10^{12}$/L)	1.435 ± 0.188	0.511	<0.001	–0.348 ± 0.334	–0.080	0.298

Abbreviations: ASMI = Appendicular skeletal muscle index.

Supplementary Table 2. Effects of multi-domain lifestyle interventions on circulating biomarker levels.

	Lifestyle Interventions					F	*p*
	**Nutritional**	**Cognitive**	**Physical**	**Combined**	**Control**		
Creatinine (μmol/L)							
Baseline,	67.07 ± 20.115	63.98 ± 15.85	71.33 ± 25.61	76.64 ± 29.61	71.62 ± 18.211	2.076	0.085
6 Month	64.89 ± 17.02	68.53 ± 18.72	68.61 ± 22.50	78.95 ± 33.07	67.91 ± 17.47	2.088	0.084
t, *p*	0.364, 0.718	2.163, 0.039	–1.012, 0.320	1.270, 0.212	–4.109, <0.001		
Irisin (ng/mL)							
Baseline	46.32 ± 58.12	38.06 ± 39.21	36.36 ± 44.20	36.13 ± 44.27	34.04 ± 48.80	0.334	0.855
6 Month	36.71 ± 37.46	44.43 ± 43.89	27.52 ± 34.67	38.72 ± 49.99	30.70 ± 41.18	0.709	0.587
t, *p*	–1.292, 0.207	1.239, 0.226	–1.087, 0.289	–0.227, 0.822	–0.575, 0.571		
CRP (μg/mL)							
Baseline	8.85 ± 22.27	6.71 ± 10.11	6.40 ± 14.37	4.86 ± 7.40	4.07 ± 7.25	0.868	0.484
6 Month	2.57 ± 2.76	2.67 ± 2.33	3.00 ± 4.51	4.11 ± 5.18	2.36 ± 3.35	1.200	0.313
t, *p*	–1.890, 0.068	–2.067, 0.048	–1.259, 0.220	–0.765, 0.449	–1.927, 0.064		
TNF–α (pg/mL)							
Baseline	9.67 ± 2.851	10.42 ± 5.821	11.09 ± 8.30	10.43 ± 4.00	9.65 ± 2.612	0.614	0.653
6 Month	9.36 ± 2.89	8.87 ± 2.75	9.96 ± 3.20	10.01 ± 3.86	9.31 ± 2.57	0.823	0.512
t, *p*	–1.290, 0.206	–1.26, 0.219	–1.203, 0.240	–2.186, 0.036	–1.126, 0.269		
GSSG (pg/mL)							
Baseline	17.62 ± 12.73	20.03 ± 21.67	22.70 ± 21.99	15.89 ± 11.32	16.18 ± 16.08	0.873	0.482
6 Month	16.71 ± 11.98	19.52 ± 15.28	21.60 ± 24.58	17.23 ± 21.54	16.55 ± 14.55	0.380	0.823
t, *p*	–0.624, 0.539	0.303, 0.764	–0.643, 0.528	0.086, 0.932	–0.404, 0.690		
DHEA–S (μg/ml)							
Baseline	86.42 ± 77.93	77.33 ± 64.08	102.69 ± 98.64	85.73 ± 94.68	83.96 ± 95.92	0.382	0.821
6 Month	86.50 ± 76.67	93.27 ± 84.61	84.15 ± 67.46	76.61 ± 72.52	81.94 ± 85.85	0.191	0.943
t, *p*	–1.090, 0.285	1.811, 0.082	–1.577, 0.129	–0.113, 0.911	0.822, 0.419		
Cortisol (μg/dl)							
Baseline	7.21 ± 2.49	7.06 ± 2.38	7.22 ± 2.50	7.00 ± 2.67	6.40 ± 2.89	0.561	0.691
6 Month	7.11 ± 2.54	7.95 ± 3.00	7.37 ± 2.56	7.23 ± 2.07	6.65 ± 2.28	0.995	0.413
t, *p*	–0.539, 0.594	1.579, 0.126	0.087, 0.931	0.491, 0.627	–0.470, 0.643		
C–peptide (ng/ml)							
Baseline	1.39 ± 0.74	1.66 ± 0.84	1.41 ± 0.61	1.63 ± 1.07	1.59 ± 0.90	0.780	0.540
6 Month	1.34 ± 0.56	1.60 ± 0.69	1.39 ± 0.69	1.78 ± 0.94	1.31 ± 0.51	2.571	0.040
t, *p*	–1.028, 0.312	–0.056, 0.955	–0.534, 0.598	0.737, 0.467	–2.121, 0.045		
Insulin (pg/ml)							
Baseline	383.26 ± 250.90	519.85 ± 270.95	417.55 ± 273.36	459.07 ± 294.20	405.06 ± 318.20	1.236	0.298
6 Month	419.07 ± 302.12	477.37 ± 207.16	416.61 ± 251.17	441.66 ± 295.79	313.01 ± 252.85	1.379	0.244
t, *p*	–0.936, 0.357	–0.812, 0.425	0.019, 0.985	–0.577, 0.568	–3.085, 0.005		
Leptin (ng/ml)							
Baseline	11.86 ± 12.21	11.68 ± 9.87	11.41 ± 10.29	11.33 ± 10.20	8089 ± 7.12	0.473	0.755
6 Month	9.60 ± 7.55	14.64 ± 13.39	9.86 ± 7.02	13.04 ± 15.59	10.89 ± 16.59	0.865	0.487
t, *p*	–1.559, 0.130	1.053, 0.302	–0.992, 0.332	0.890, 0.380	–0.903, 0.376		
Haemoglobin (g/dL)							
Baseline	13.34 ± 1.11	12.93 ± 1.14	13.25 ± 1.47	13.26 ± 1.66	13.51 ± 1.26	1.105	0.355
6 Month	13.38 ± 1.17	13.01 ± 1.20	13.46 ± 1.51	13.53 ± 1.54	13.20 ± 1.32	0.867	0.485
t, *p*	–1.030, 0.312	–0.585, 0.563	0.025, 0.981	–0.472, 0.640	–0.275, 0.786		

Haematocrit (%)							
Baseline	40.12 ± 3.24	38.82 ± 3.19	40.00 ± 3.74	40.26 ± 3.89	40.95 ± 3.69	1.751	0.141
6 Month	40.36 ± 3.61	39.18 ± 3.15	41.66 ± 4.36	41.23 ± 3.90	40.33 ± 3.63	1.566	0.188
t, *p*	1.233, 0.231	0.620, 0.541	–0.854, 0.408	1.061, 0.299	–1.056, 0.302		
Red blood cell ($\times 10^{12}$/L)							
Baseline	4.54 ± 0.38	4.44 ± 0.33	4.47 ± 0.34	4.54 ± 0.42	4.59 ± 0.42	0.840	0.501
6 Month	4.50 ± 0.34	4.49 ± 0.41	4.60 ± 0.35	4.60 ± 0.35	4.54 ± 0.34	0.529	0.714
t, *p*	–0.264, 0.794	1.783, 0.092	0.332, 0.743	–0.653, 0.522	–1.660, 0.111		

Data are presented as mean ± SD. Abbreviations: CRP = C-reactive protein, TNF-α = tumor necrosis factor alpha, DHEA-S = dehydroepiandrosterone sulfate.
